# Microscopy analysis and production rate data for needleless vertical rods electrospinning parameters

**DOI:** 10.1016/j.dib.2015.08.005

**Published:** 2015-08-20

**Authors:** Hyeon Ung Shin, Yalong Li, Ariel Paynter, Kitchaporn Nartetamrongsutt, George G. Chase

**Affiliations:** Department of Chemical and Biomolecular Engineering, The University of Akron, OH 44325, USA

**Keywords:** Electrospinning, Polymer fibers, Electric fields

## Abstract

A multiple vertical rod setup for needless electrospinning was used to fabricate submicron polymer fibers. The design with multiple vertical rods is a new concept for increased production of electrospun fibers. Different geometries and operating conditions are possible. The effects of varying the number of rods in the array have been studied and reported [1]. The goal of this work was a proof of concept of the threaded rod design by exploring the effects of variations in applied voltage and gap distance for a fixed array of rods. Effects on fiber diameter and production rate of fibers are reported. More extensive experiments are needed to quantify the interrelations between parameters and to guide the design and operation of the method. No attempt was made to optimize the operating parameters or the geometry in terms of production rates or fiber diameters.

## Specifications table

**Table:** 

Subject area	*Physics, Chemistry.*
More specific subject area	*Needleless electrospinning.*
Type of data	*Figure, Images* (*scanning electron microscopy*).
Howdatawasacquired	*Fiber size distributions were determined from SEM images and their analysis using Fibraquant 1.3 software* (*Nanoscaffold Technologies LLC*); *Production rates were determined by measuring the mass of fibers collected per unit time.*
Data format	*Analyzed, tabulated and plotted.*
Experimental factors	*6*% *PVP polymer was dissolved in ethanol to electrospin. Gap distances and applied voltages were varied.*
Experimental features	*Vertical rod spinning parameters* (*gap distance and voltage*) *affected the fiber size distributions and production rates of electrospun PVP fibers.*
Data source location	*University of Akron, Akron, Ohio, USA.*
Data accessibility	*Data is provided with this article.*

## Value of the data

**Table:** 

• The electrospun fiber production rates experimentally increased as the applied voltage increased. The changes in fiber size and production rates shown to be statistically significant using a one-way ANOVA analysis. • The fiber diameter decreased as the distance between the collector and the plane of the rod arrays increased from 15.2 to 35.6 cm. • The increase of distance between collector and electrically charged linear array of vertical rods significantly reduced the production rate to 0.005 g/min and was shown to be statistically significant verifies its significance in affecting the production rate.

## 1 Data, experimental design, materials and methods

**Fig. 1:**
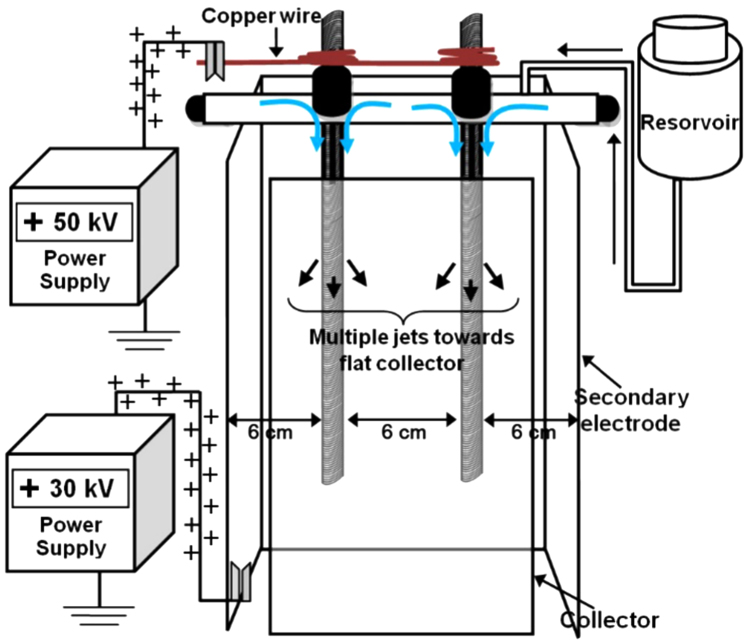
Array with two vertical rods. The arrows show the general directions of the jets.

Experimental details are described in reference [1]. The data presented here are for a vertical rod array geometry of 2 rods positioned in a planar linear array positioned parallel to a flat collector and backed by a secondary electrode. The rods were 50 cm long, spaced 6 cm apart, and the secondary electrode was 6 cm behind the plane of the rods. The secondary electrode had wings on each end also spaced 6 cm from the nearest rod. The geometry of the setup is shown in Fig. 1.

A 6 wt% solution of Polyvinylpyrrolidone (PVP, Aldrich, *MW*: 1,300,000) was prepared by dissolving PVP in ethanol (AAPER alcohol, 200 proof). It was used for fabrication of submicron-sized PVP fibers by a linear array of 2 vertical rods electrospinning setup. Secondary electrodes were positioned on one side of the linear array rods to direct the jets toward a planar collector surface (1680 cm^2^).

In this data, the effect of applied voltage and gap distance between vertical rod and planar collector was investigated to compare the fiber morphology, diameter and production rate. Applied voltages are typically in the 10–50 kV range for electrospinning. In trial experiments it was determined that adequate electrospinning occurred with voltages above about 25 kV hence the experiments here considered 30, 40 and 50 kV. Also, typical gap distances range from 10 to about 30 cm in literature. We chose to conduct experiments at three distances starting at 15 cm and increasing to about 35 cm.

**Fig. 2:**
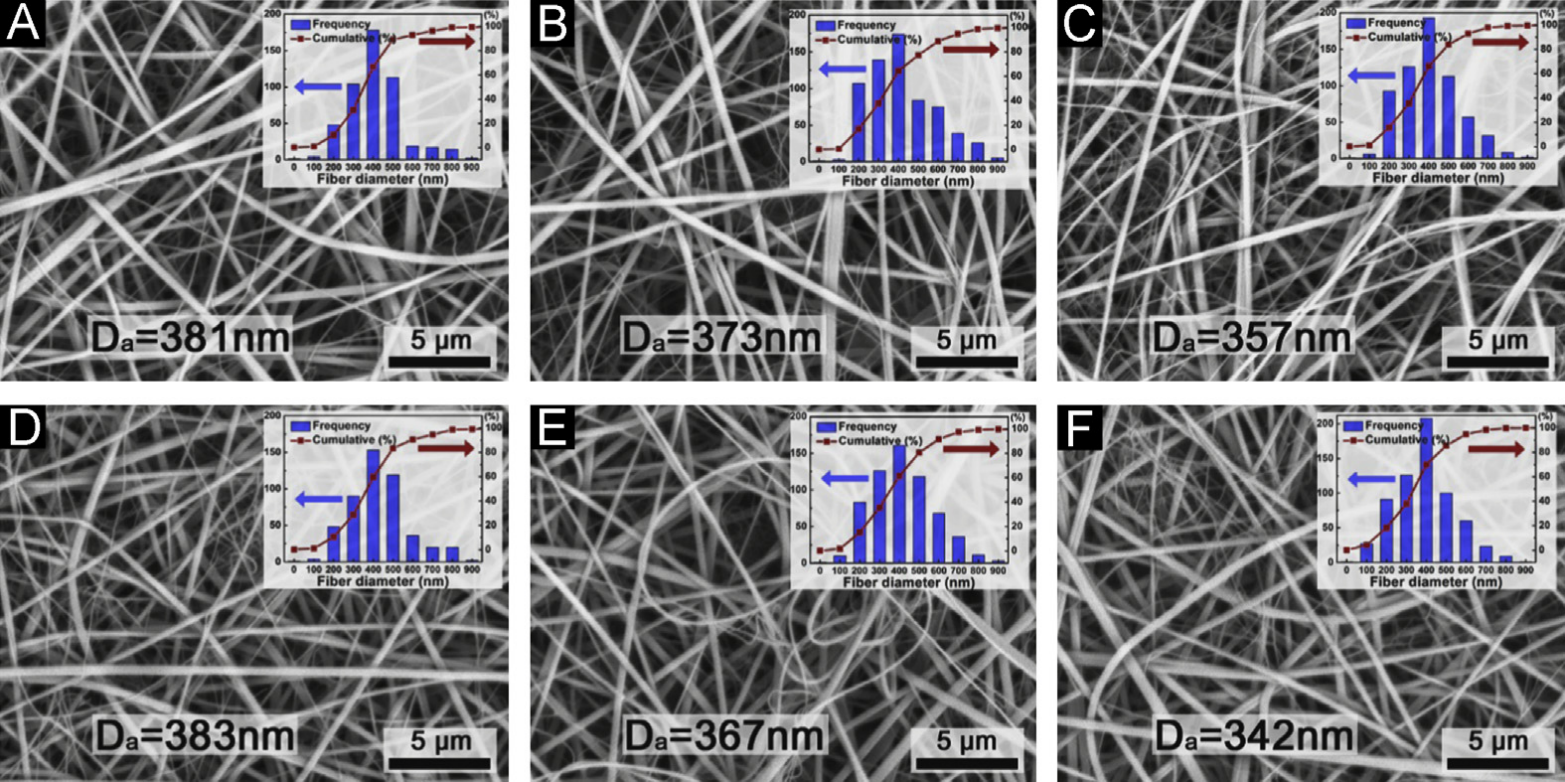
SEM images of electrospun PVP fibers with variation of applyied voltages: 30 kV (A), 40 kV (B), and 50 kV (C) all with a 15.2 cm gap distance. SEM images of electrospun PVP fibers with variation of gap distance 15.2 cm (D), 25.4 cm (E), and 35.6 cm (F) all with an applied voltage of 50 kV.

Fig. 2(A)–(C) shows the electrospun fibers with variation of the applied voltages at 30 kV (A), 40 kV (B), 50 kV (C) and a 15.2 cm gap distance. The morphologies of the fibers were continuous and without beads. The diameter distributions were in the similar size range of 200–800 nm with 381 nm (30 kV), 373 nm (40 kV), 357 nm (50 kV) of average diameter. Furthermore, the production rate gradually increased with increase of the applied voltage. These results are in good agreement with literature where high voltages and high electric field strengths are associated with higher productivity [2–5].

To prove the statistical significance and cause and effect between applied voltage and size distribution of fibers, a one-way ANOVA analysis was performed on the data. With regard to our test, the applied voltage was the regressor variable *X* and fiber size distribution is the response variable *Y* with an assumed linear relationship *Y*=*β*_1_*X*. As the result, the *P*-value of hypothesis was computed as 0.045 which is less than the selected *α*=0.05, which rejects the null hypothesis that *H*_*0*_: *β*_1_=0 in terms of the linear relationship and therefore validates that the applied voltage for electrospinning had a significant impact on the fiber size distribution.

Fig. 2(D)–(F) shows the fiber diameter distributions with respect to changes of gap distance 15.2 cm (D), 25.4 cm (E), and 35.6 cm (F) with a fixed voltage of 50 kV. It is observed that the gap distance directly affected the average fiber size. With increasing the collecting distance from the vertical rods, the fiber size decreased to 342 nm due to increased time and distance for the jet to stretch in flight to the collector.

**Fig. 3:**
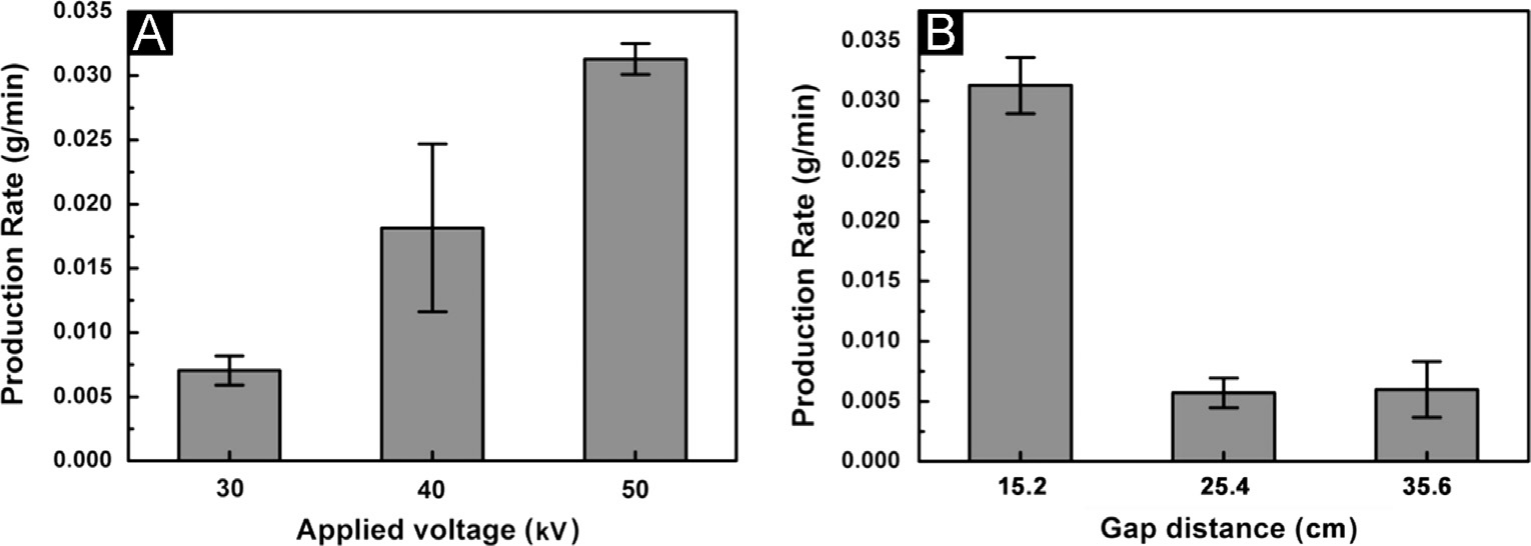
(A) Production rate versus applied voltage at 15.2 cm gap distance. (B) Production rate versus gap distance at applied voltage of 50 kV.

Fig. 3 shows the effects of applied voltage and gap distance on the production rate of fibers. As the applied voltage increased the production rate increased almost proportionately (Fig. 3(A)). Production rate was high for the smallest gap distance of 15.2 cm. Production rates dropped significantly for the 25.4 and 35.6 cm gaps (Fig. 3(B)) This is in good agreement with literature [6–9].

The influence of gap distance on production rate of electrospun fiber is also investigated by the one-way ANOVA analysis to provide statistical validation on observation results. The production rates of 15.2, 25.4, and 35.6 cm gap distance are used as response variables in the test for significance of regression with *P*-value calculated. Since the resultant *P*-value 4.8E−6 is considerably smaller than *α*=0.05, similarly by means of the abovementioned analysis, the test substantiates the significance of gap distance as regressor variable in the model and statistically confirms that the production rates vary with change of distance between the electrode and collector area.

## Conflict of interest

None.
